# Commensal Rodent Habitat Expansion Enhances Arthropod Disease Vectors on a Tropical Volcanic Island

**DOI:** 10.3389/fvets.2021.736216

**Published:** 2021-10-08

**Authors:** De-Lun Wu, Han-Chun Shih, Jen-Kai Wang, Hwa-Jen Teng, Chi-Chien Kuo

**Affiliations:** ^1^Department of Life Science, National Taiwan Normal University, Taipei, Taiwan; ^2^Epidemic Intelligence Center, Centers for Disease Control, Ministry of Health and Welfare, Taipei, Taiwan; ^3^Center for Diagnostics and Vaccine Development, Centers for Disease Control, Ministry of Health and Welfare, Taipei, Taiwan

**Keywords:** rodent, invasive species, volcanic island, scrub typhus, *Orientia tsutsugamushi*, vector-borne diseases, island syndrome, *Rattus tanezumi*

## Abstract

On volcanic islands, the release of animals from predators and competitors can lead to increased body size and population density as well as the expanded habitat use of introduced animals relative to their mainland counterparts. Such alterations might facilitate the spread of diseases on islands when these exotic animals also carry pathogenic agents; however, this has rarely been investigated. The commensal Asian house rat (*Rattus tanezumi*) is confined to human residential surroundings in mainland Taiwan but can be observed in the forests of nearby Orchid Island, which is a tropical volcanic island. Orchid Island is also a hot spot for scrub typhus, a lethal febrile disease transmitted by larval trombiculid mites (chiggers) that are infected primarily with the rickettsia *Orientia tsutsugamushi* (OT). We predicted an increase in chigger abundance when rodents (the primary host of chiggers) invade forests from human settlements since soils are largely absent in the latter habitat but necessary for the survival of nymphal and adult mites. A trimonthly rodent survey at 10 sites in three habitats (human residential, grassland, and forest) found only *R. tanezumi* and showed more *R. tanezumi* and chiggers in forests than in human residential sites. There was a positive association between rodent and chigger abundance, as well as between rodent body weight and chigger load. Lastly, >95% of chiggers were *Leptotrombidium deliense* and their OT infection rates were similar among all habitats. Our study demonstrated potentially elevated risks of scrub typhus when this commensal rat species is allowed to invade natural habitats on islands. Additionally, while the success of invasive species can be ascribed to their parasites being left behind, island invaders might instead obtain more parasites if the parasite requires only a single host (e.g., trombiculid mite), is a host generalist (e.g., *L. deliense*), and is transferred from unsuitable to suitable habitats (i.e., human settlements on the mainland to forests on an island).

## Introduction

It is well-known that biota on isolated oceanic islands are particularly vulnerable to the invasion of exotic species compared with their continental counterparts. Most oceanic islands are depauperate in terrestrial biota relative to mainland areas due to the difficulty experienced by species in colonizing isolated islands. This renders insular species less exposed to predators and competitors and evolve less defensive capabilities (1). Moreover, a small land area leads to a small population size, which increases the likelihood of species extinction. Therefore, biological invasions can have a devastating effect on island biodiversity (2). For example, the introduction of a predatory snail to biologically control the invasive giant African snail has exterminated most endemic tree snail species in French Polynesia (3). Bird species endemic to islands are more prone to extinction than continental species, which is largely due to the detrimental effects of mammalian predators such as rats, cats, and pigs, among others (4).

**Figure 1:**
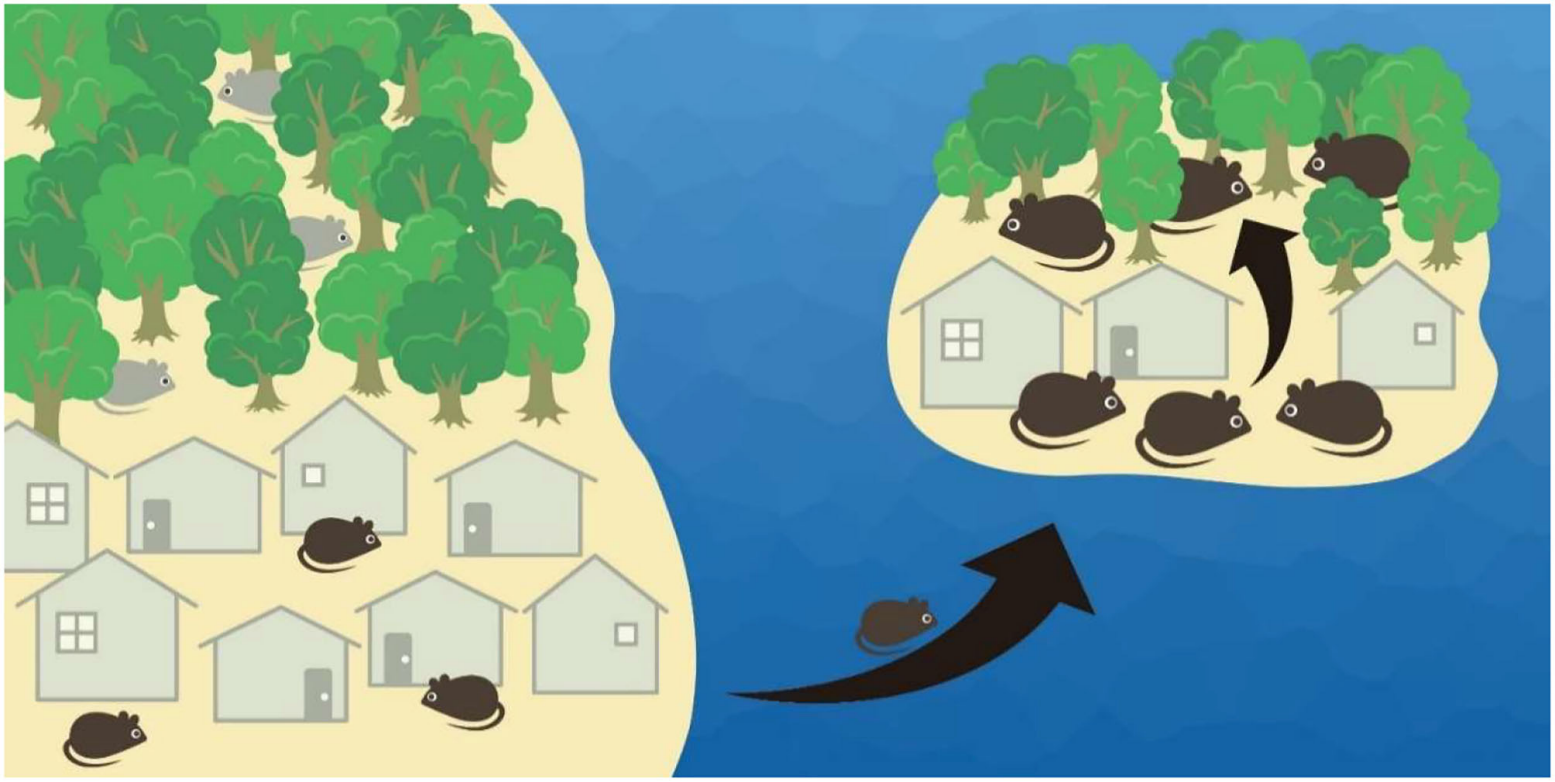
When colonizing ocean islands from the mainland, rodents generally become larger and reach higher population densities. Also, release from competitors on islands allows rodents to expand habitat use; e.g., commensal rodent species (black color) is confined to human settlements on the mainland when competitive species (gray color) occupies the forests, but is allowed to enter the forests when the competitor is absent on the island.

In contrast, little attention has been paid to whether oceanic islands can also facilitate the spread of diseases when exotic species also happen to carry pathogens or arthropod disease vectors. For example, rodents are common island invaders (5, 6) and also host many vector-borne and zoonotic diseases (7, 8). At least three mechanisms can aid the spread of disease on islands. First, ecological release from interspecific competition on islands with low species diversity can facilitate the habitat expansion of colonizers (9, 10) (Figure 1). The meadow vole occupies open fields on the mainland but extends to woodlands on islands when the woodland-inhabiting deer mouse occurs in the former but not the latter (11). Therefore, disease-carrying invaders likely originating from human-disturbed environments where most exotic species are introduced and habituate to (12, 13) can spread diseases following habitat expansion to more pristine habitats (e.g., forests) on oceanic islands.

Second, rodents often attain higher population density on islands than in mainland habitats (11, 14, 15) (Figure 1). Due to the so-called “island syndrome,” such systematic differences in demography are thought to be a direct effect of limited dispersal for insular populations (i.e., the fence effect) as well as an indirect outcome of small land area that releases island species from fewer predators and competitors (14). On the other hand, the transmission of many pathogens is density dependent, with epidemiological models predicting that an elevated density of susceptible host populations can increase the contact rate and help sustain disease transmission (16). For example, the Sin Nombre virus was less detected following rodent population decline in the southwestern United States (17); however, other correlation studies between host density and hantavirus prevalence do not detect a clear pattern (18). Therefore, higher population density on oceanic islands could potentially facilitate disease transmission.

Lastly, rodent body size is usually larger on small islands than in mainland habitats (14, 19) (Figure 1). Such gigantism could be the result of higher intraspecific competition following high population density on islands, which favors increased life span and body mass (14). This could also be due to the advantage of a larger body size in securing food resources when a small body size (for escaping predation) is not selected for on predator-free islands (20). At the same time, it has been found that the burden of some disease vectors (e.g., ticks) would increase with the body mass of hosts (e.g., rodents) (21–23). This is likely the result of larger surface areas for ectoparasites to attach to (24) or larger hosts being more tolerant to parasite infestations (25, 26). Consequently, the increased body size of hosts on oceanic islands could presumably maintain a higher number of vectors and thus help sustain vector-borne diseases.

Orchid Island, also known as the Lanyu Island, is a tropical volcanic island with an area of 46 km^2^ that lies 76 km off the southeastern coast of Taiwan. Between 1991 and 2020, the annual mean temperature was 22.8°C (monthly range: 18.6–26.2°C) and yearly rainfall totaled 2,979 mm (Taiwan Center Weather Bureau, https://www.cwb.gov.tw/V8/C/, accessed March 10, 2021). More than 80% of Orchid Island remains covered with forests, which are mostly (ca. 80%) primary forests (27). However, a high abundance of the commensal Asian house rat (*Rattus tanezumi*) was observed in the forests and grasslands of this island (28). This situation differs from the main island of Taiwan and other parts of Southeast Asia, where *R. tanezumi* is largely restricted to areas surrounding human settlements (29). On the main island of Taiwan, lowland forests and grasslands are instead occupied by the native *Niviventer coninga* and *Rattus losea*, respectively (30).

Orchid Island is also a hot spot for scrub typhus. Scrub typhus is an acute and deadly infectious disease transmitted by larval trombiculid mites infected primarily with the rickettsiae *Orientia tsutsugamushi* (OT). Previously confined to the western Pacific, southern Asia, and northern Australia (31), exposure to this bacterium has recently been observed in South America (32, 33), the Middle East (34), and Africa (35–37). Simultaneously, several countries have experienced a great increase in human incidences of scrub typhus (38–40). The life cycle of trombiculid mites includes the egg, larva, nymph, and adult stages. Only the larval stage is parasitic, with rodents as the primary hosts (41–43). Notably, mites at this stage are commonly called chiggers. In contrast, the nymphal and adult stages live freely in soil and consume arthropods (43). The efficient transovarial and transstadial transmission of OT occurs in trombiculid mites, which are reservoirs of OT, while animal hosts (e.g., rodents) play no role in transmitting OT (43). In eastern Taiwan where scrub typhus is already severe (44), Orchid Island has the highest disease prevalence among local districts (45, 46). On Orchid Island, the antibody positivity rate for children at 5 and 6 years old is ~60 and 70–80%, respectively, but became 100% when children reached 7 years old (47). Recently, an international traveler to Orchid Island died of scrub typhus (48).

Here, we investigated whether the habitat expansion of the commensal Asian house rat would increase the risk of scrub typhus in extended habitats. Specifically, we hypothesized that chiggers would increase when house rats expanded from human settlements to forests. This is due to the life cycle of trombiculid mites being interrupted by paved ground in the former habitat, where the soils necessary for nymphal and adult mites to survive are largely absent. In addition, although it has long been emphasized that scrub typhus can occur in a broad range of habitats other than scrub habitat (particularly forests) (42), the relative suitability of forests for sustaining scrub typhus has never been quantified. Lastly, despite Orchid Island being a hot spot of scrub typhus, no systematic surveillance of chigger vectors has ever been attempted on this island; it was only briefly surveyed as part of a nationwide investigation on vectors of scrub typhus (49). Nevertheless, a good knowledge of chiggers is helpful for the prevention and control of scrub typhus on Orchid Island.

## Materials and Methods

### Study Sites

**Figure 2:**
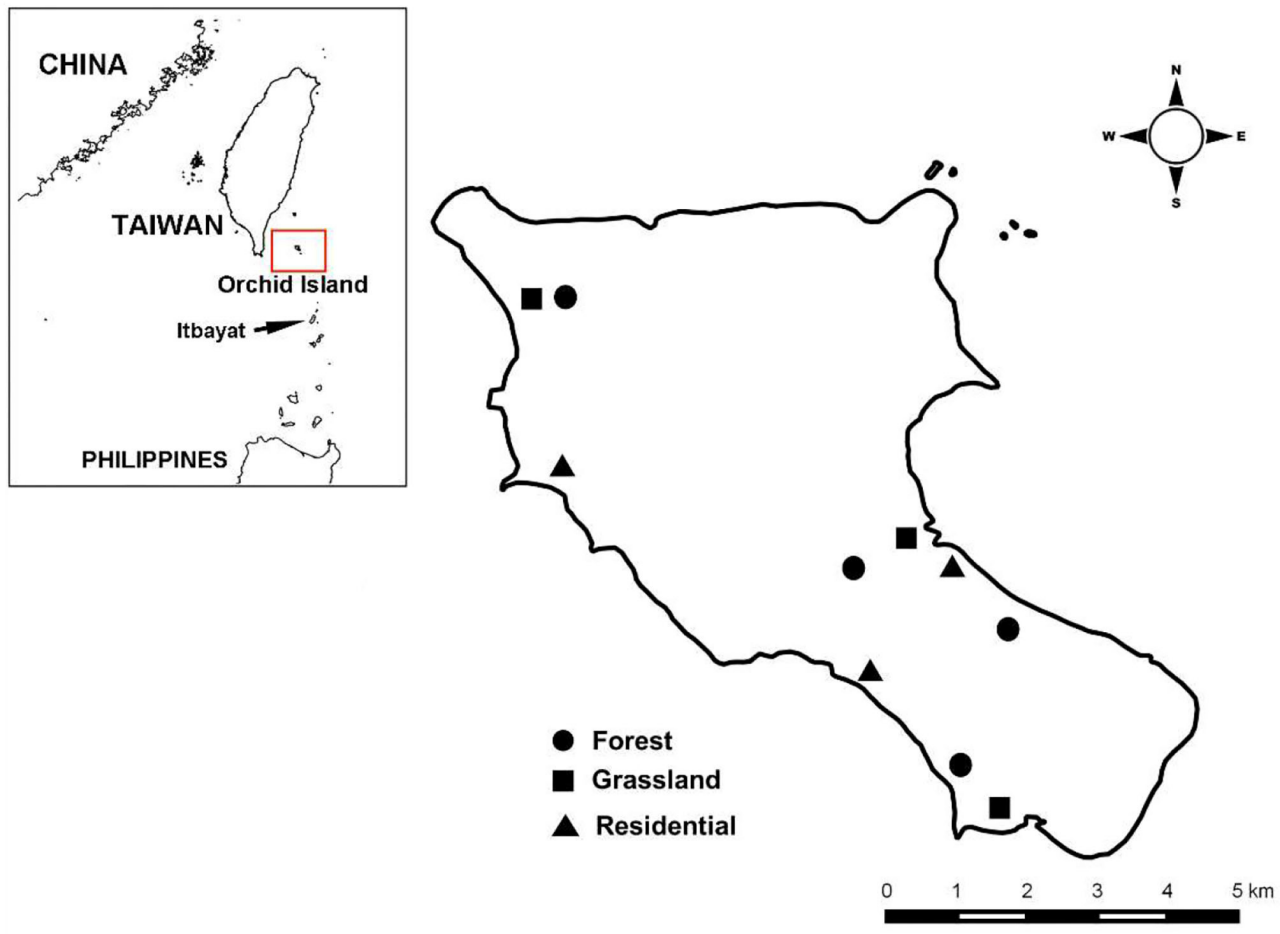
Study sites on Orchid Island of Taiwan.

We surveyed rodents and associated chiggers at 10 sites in three dominant habitat types (50) on Orchid Island (Figure 2; Figure S1 for satellite image). This included three human residential sites, three grassland sites, and four forest sites. Sites of the same habitat type were located in different regions of the island to control for potential regional difference in rodent and chigger abundance. Distances between sites were >500 m, except for two sites located in the northwest of the island (distance: 250 m). Distances from sites to ecotones were >200 m except for the two abovementioned sites (distance: 100 m). The maximum home range size for radio-tracked *R. tanezumi* on Orchid Island was 0.27 ha (equal to a radius of 29.3 m) and 0.37 ha (34.3 m) for males and females, respectively (29). Therefore, we expected that trapped rodents would spend the majority of their time in the designated sites and habitats. Residential sites are characterized by paved ground houses and roads surrounded by plant species adapted to human disturbance. Grasslands are dominated by silver grass (*Miscanthus sinensis*), while forests are composed of broadleaf species, mainly *Bischofia javanica, Ficus benjamina, Garcinia linii, Palaquium formosanum*, and *Pometia pinnata*, among others.

### Small Mammal Trapping and Collection of Chiggers

From September 2017 to June 2018, small mammal traps were set up in each of the 10 study sites every 3 months. In each site, Sherman traps (26.5 × 10.0 × 8.5 cm) and mesh traps (27 × 16 × 13 cm) were deployed alternatively every 10–15 m along a transect line for a total of 20 traps (each with 10 traps). Traps were baited with sweet potatoes smeared with peanut butter and were left open for three consecutive nights. For each seasonal trapping session, 10 sites were surveyed within 10 days to avoid any confounding temporal influence on rodents and chiggers.

Once trapped, small mammals were transferred to clean nylon bags and species were identified. Since all of the trapped rodents were the exotic Asian house rat, they were not released back to the field. This might reduce rodent population size; however, since the main purpose of this study was to compare habitats (instead of obtaining an accurate population size), and rodents were removed from all sites instead of only in certain habitats, the effects of such rodent removal on our comparative study should be negligible. An overdose of 0.1 ml of Zoletil 50 anesthetic (Virbac, Carros, France) was injected subcutaneously, followed by cervical dislocation to euthanize sedated rats. The sex and reproductive status of each rodent were determined, and the body, tail, ear, and hind foot lengths (mm) were measured along with body mass (g). We checked for the presence of ectoparasites by thoroughly examining the entire body of the animal with the naked eye. Ears and other body parts with chiggers attached were detached and placed in vials. Chiggers were allowed 1 day to release themselves from rats before being preserved in 75% ethanol. We stored chiggers at −20°C for subsequent molecular determination.

### Chigger Identification

Due to the large number of collected chiggers (>100,000), only 1/10th of the chiggers from each rodent was randomly retrieved for species identification. Chiggers were soaked in deionized water two to three times to replace ethanol with water and then slide-mounted in Berlese fluid (Asco Laboratories Ltd., Manchester, UK). Chiggers were then examined under an upright microscope (Olympus BX53, Tokyo, Japan) and identified to the species level by following published keys (51–53).

### OT Detection in Chiggers

Due to the small size of chiggers, 30 chiggers of the same species from the same individual rat were pooled to retrieve sufficient DNA for the detection of OT. This also allowed the potential comparison of the infection rate of OT in previous studies that pooled the same number of chiggers [e.g., (54, 55)]. We followed (54) by using the nested polymerase chain reaction to target the well-conserved 56-kDa type-specific antigen located on the OT outer membrane. Laboratory OT strains and nuclease-free water were used as positive and negative controls, respectively.

### Statistical Analysis

The difference in trapping success between the two trap types, as well as the difference in prevalence of OT infection in chiggers among habitats and months, was assessed with chi-square test. Generalized estimating equations (GEE) were applied to compare the number of rodents and their total infested chiggers in each sampling site among habitats and months. Habitat, month, and the interactions of both factors were the fixed factors, with the site as the subject and each seasonal sampling as repeated measures within the site (10 sites × 4 sampling sessions = 40 samples overall). A normal distribution function and negative binomial log link function were used for rodent and chigger abundance, respectively. The significance of the difference was determined based on a 95% Wald confidence interval of estimated marginal means.

A generalized linear mixed model (GLMM) was used to analyze how the chigger load of individual rodents varied with habitat, month, and body size. Habitat, month, and the interactions of both factors, as well as rodent body weight, were the fixed factors, while site was a random factor. Similarly, a negative binomial link function was used and the significance of the difference was determined based on the 95% Wald confidence interval of estimated marginal means.

All the analyses were performed in SPSS Statistics version 19.0 (IBM Corp.) and the lme4 package in R 3.6.1.

## Results

### Rodent Abundance Across Habitats and Months

A total of 254 rodents were captured over 2,400 trap-nights, with a trapping success of 10.6% (number of rodents per trap-night). Only 9 out of the 254 rodents were trapped using Sherman traps (trapping success 0.8%), while the remaining 245 rodents were trapped using mesh traps (20.4%). There was a significant difference in trapping success between the two trap types (χ^2^ = 243.2, *df* = 1, *p* < 0.001). The total number of rodents trapped in forests, grasslands, and residential sites was 148, 71, and 35, respectively. All of the captured rodents were Asian house rats (*R. tanezumi*).

**Figure 3:**
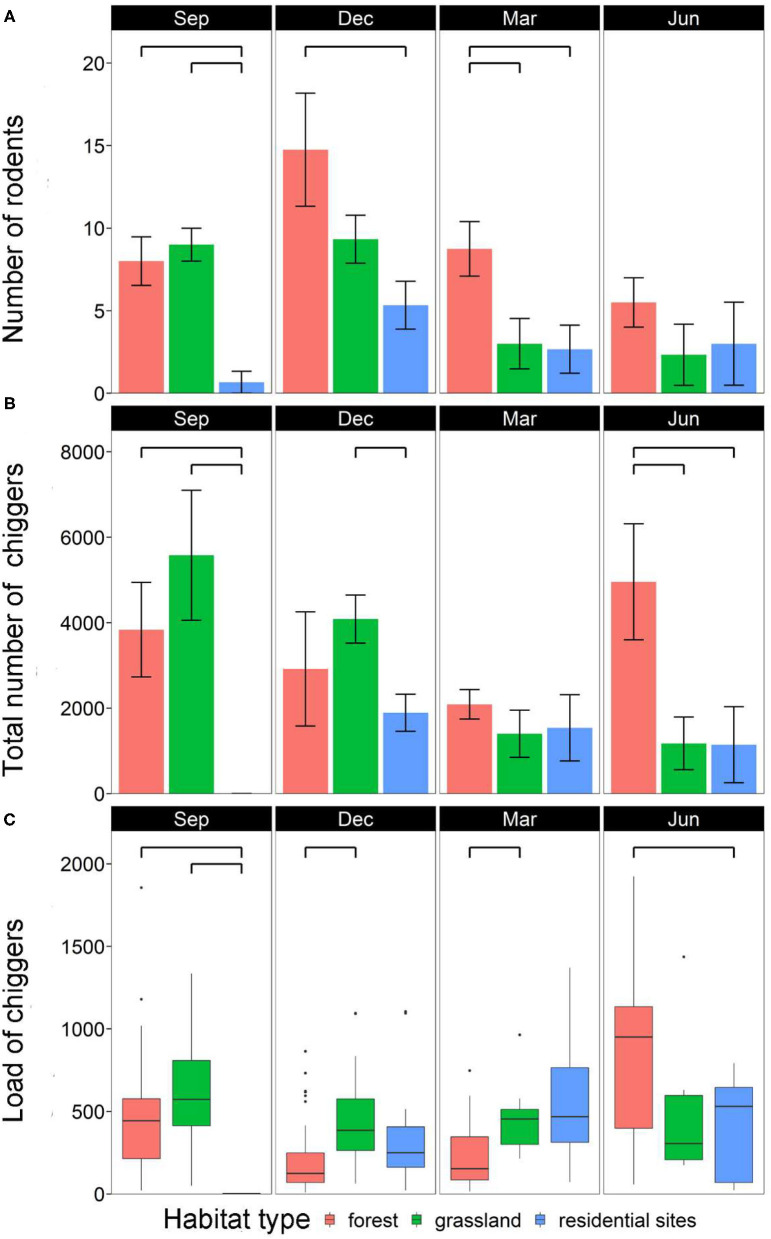
Monthly variations among habitats in **(A)** mean number of rodents per study site, **(B)** mean number of total chiggers per study site, and **(C)** chigger load per rodent individual. Each bar is represented by mean ± SE; significant difference (*p* < 0.05) between two groups is annotated with a bridge.

Rodent abundance varied among habitats (χ^2^ = 20.5, *df* = 2, *p* < 0.001) and months (χ^2^ = 22.2, *df* = 3, *p* < 0.001), and there was an interaction between habitats and months (χ^2^ = 50.6, *df* = 6, *p* < 0.001). There were significantly more rodents in forests than in residential sites for most of the months (except for June) (*p* < 0.05). By contrast, rodent abundance was largely similar between forests and grasslands (except for March) and between grasslands and residential sites (except for September) (*p* > 0.05) (Figure 3A).

### Total Chiggers Across Habitats, Months, and Association With Rodent Abundance

A total of 105,680 chiggers were collected, and all rodents were found infested with chiggers (prevalence = 100%). However, the total number of chiggers per site varied among habitats (χ^2^ = 126.5, *df* = 2, *p* < 0.001) and months (χ^2^ = 50.4, *df* = 3, *p* < 0.001), and there was an interaction between habitats and months (χ^2^ = 3,775.3, *df* = 6, *p* < 0.001). There were significantly more chiggers in forests than in residential sites in September and June (both *p* < 0.05). There were also more chiggers in grasslands than in residential sites in September and December (both *p* < 0.05). The abundance of chiggers was similar in forests and grasslands except for in June (*p* > 0.05) (Figure 3B).

**Figure 4:**
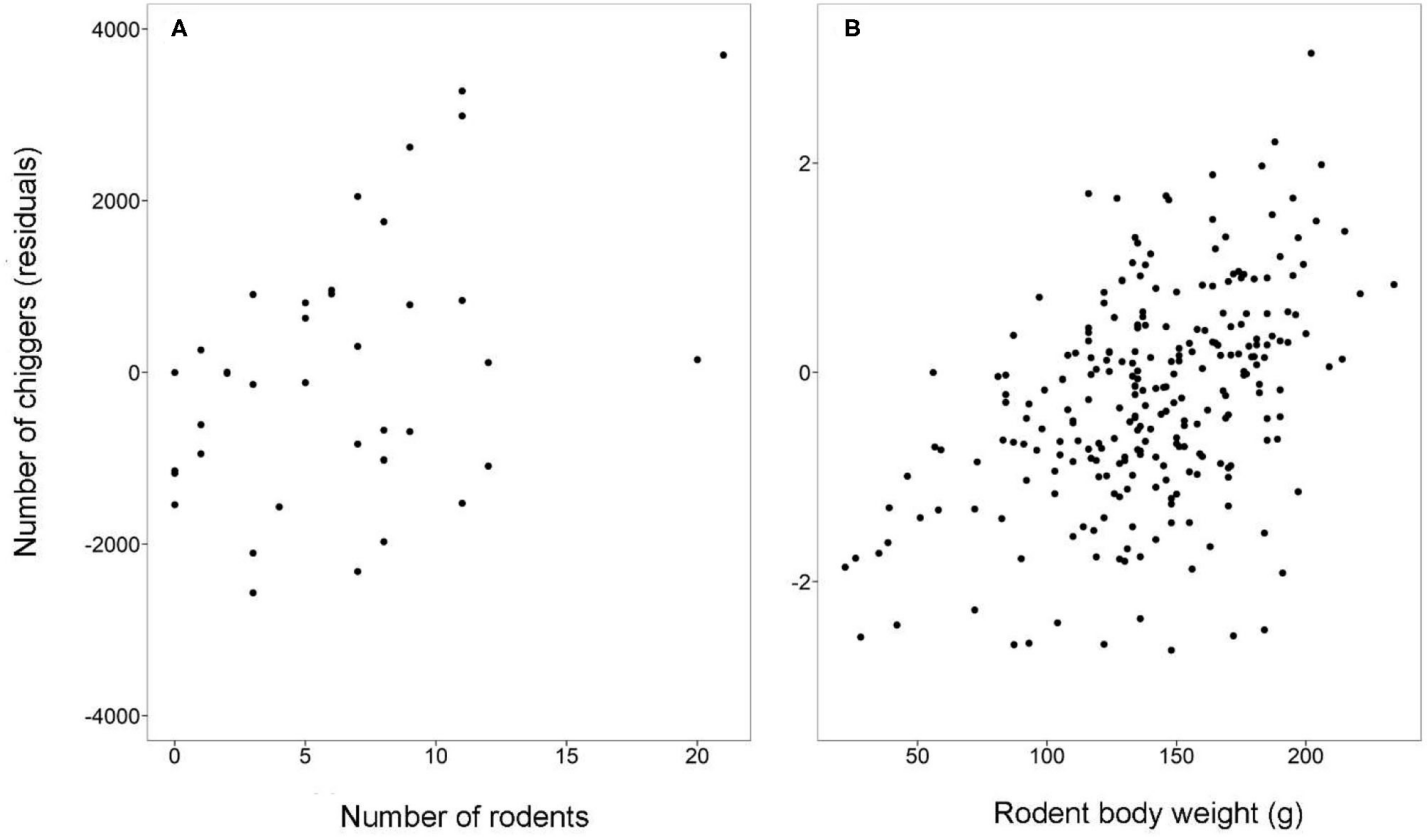
Scatterplots of the number of chiggers (residuals, controlled for effect of habitat, month, and their interactions) against **(A)** the number of rodents per study site and **(B)** rodent body weight. Both scatterplots show significantly positive associations (see the Results).

To further explore whether the difference in chiggers was the direct effect of habitats and months or indirectly mediated through their effect on rodent abundance (as per the previous section), rodent abundance was included in the above model as a covariant. Results showed that in addition to the direct effect of habitats, months, and their interactions on total chiggers (χ^2^ = 70.9, 114.0, 2,458.4, respectively, all *p* < 0.001), rodent abundance was also positively associated with chigger abundance (χ^2^ = 51.6, β = 0.18, *p* < 0.001) (Figure 4A). Therefore, habitats and months both directly and indirectly affected chigger abundance, which was mediated by their effect on rodent abundance.

### Chigger Load Across Habitats, Months, and Association With Rodent Body Weight

The chigger load of individual rodents varied among habitats (χ^2^ = 11.9, *df* = 2, *p* < 0.005) and months (χ^2^ = 80.6, *df* = 3, *p* < 0.001), and there was an interaction between habitats and months (χ^2^ = 83.2, *df* = 6, *p* < 0.001). Chigger load was also positively associated with rodent body weight (χ^2^ = 88.8, *df* = 1, *p* < 0.001) (Figure 4B). Rodents had higher chigger load in grasslands than in forests in December and March (both *p* < 0.05). Chigger load was also higher in grasslands than in residential sites in September (*p* < 0.05). Additionally, chigger load was higher in forests than in residential sites in September and June (both *p* < 0.05) (Figure 3C).

### Species Composition of Chiggers

We morphologically identified 10,368 chiggers to the species level. We excluded 208 chiggers that could not be reliably identified (due to damaged body parts or inadequate specimen placement). The remaining 10,160 chiggers included 9,712 *Leptotrombidium deliense* (95.6%), 341 *Walchia xishaensis* (3.4%), and 107 *Eutrombicula wichmanni* (1.1%). *L. deliense* was observed in all habitat types across all sampling months. *W. xishaensis* mainly occurred in forests in September and June, while *E. wichmanni* was found primarily in a residential site in northwest Orchid Island during March and June.

### Prevalence of OT Infection in Chiggers Among Chigger Species, Habitats, and Months

A total of 200 pools of chiggers were assayed for OT infection, including 22 pools of *W. xishaensis* and 178 pools of *L. deliense*. OT was not detected in any of the *W. xishaensis* pools (0% prevalence), while the prevalence of infection in *L. deliense* pools was 37.1%.

The infection prevalence of *L. deliense* pools was similar in residential (23.3%, *N* = 30 pools), grassland (37.1%, 62 pools) and forest sites (41.9%, 86 pools) (χ^2^ = 2.6, *df* = 2, *p* > 0.05). Likewise, there was no seasonal difference in prevalence among September (37.5%, 40 pools), December (46.8%, 62 pools), March (35.6%, 45 pools), and June (19.4%, 31 pools) (χ^2^ = 6.7, *df* = 3, *p* > 0.05).

## Discussion

In comparison with residential sites, forests harbored more rodents and chiggers. In addition, the infection prevalence of OT in *L. deliense* chiggers—the most important scrub typhus vector species in Southeast Asia (43) and also the primary vector species on Orchid Island—in forests was 1.8 times that of residential sites (41.9% vs. 23.3%). We also found a positive association between rodent body weight and chigger load, as well as between rodent abundance and the total number of chiggers.

On Orchid Island, the expansion of commensal *R. tanezumi* from residential sites to forests and grasslands has helped the current spread of chiggers and thus scrub typhus infection risks from human settlements to these two natural habitats. Extinct rodent species on Orchid Island have not been studied and their influence (if any) on ancient risks to scrub typhus cannot be evaluated. *R. losea*, a native species in Taiwan, has been infrequently trapped on Orchid Island before and only occurred in grasslands (28). *R. losea* is a terrestrial (instead of arboreal) species living predominantly in grasslands, agricultural fields, and human settlements that rarely enters forests (56). Likewise, *Bandicota indica* (native) and *Rattus norvegicus* (exotic) have scarcely been recorded on this island [review by (28)]. These two species have more specialized habitat use than *R. tanezumi* (57) and live primarily in agricultural fields and human settlements, respectively. Therefore, the high flexibility of *R. tanezumi* in habitat use (57) could make this species more likely to thrive in forests than the other candidate species (i.e., *R. losea, R. norvegicus*, and *B. indica*). *R. tanezumi* has occasionally been observed in trees (personal observations), which suggest that they are also capable of adopting an arboreal lifestyle.

The effect of *R. tanezumi* habitat expansion is particularly prominent in forests since they harbor a much greater number of infected chiggers than residential sites. Forests had more rodents than the other two habitats. Moreover, larger differences were observed during the winter and early spring (December and March, Figure 3A) when the monsoon brought high winds and low temperatures, and food resources were presumably more scarce. With their complex stand structures and abundant tree species that bear large fruits (e.g., *P. formosanum* and *P. pinnata*), the forests could provide rodents with a better shelter.

Simultaneously, forests also harbored significantly more chiggers [with all months pooled, forests (mean number of chiggers per site: 3,282) had more chiggers than grassland (2,475), in turn more than residential sites (273), both *p* < 0.05]. More strikingly, we observed a great increase in chigger abundance in June when chiggers in the other habitats were in decline (Figure 3B). Both higher rodent numbers and intrinsic differences in habitat characteristics have contributed to more chiggers in forests. Higher rodent abundance should increase the probability of host finding by questing chiggers that are vulnerable to desiccation (42, 43, 58). Furthermore, forests can also maintain higher soil humidity with their closed canopies. Moreover, tropical forest soils are rich in arthropods (59, 60), which are the required food for nymphal and adult mites. Our study not only validates the occurrence of scrub typhus in forests (42) but also further demonstrates forests as a better habitat for chiggers.

On the other hand, the habitat difference in chigger load is more varied (Figure 3C). In most months, chigger loads were not always lower in residential sites than in the other two habitats. For example, the mean chigger load in March was much higher (although not significantly higher) in residential sites than in forests. This can be due to a greater abundance of rodents in the latter habitat (Figure 3A) resulting in the chiggers therein being less concentrated among rodent hosts. Overall, total chigger abundance remained higher in the forest (Figure 3B). Unpaved areas surrounding human settlements might help sustain the chigger population. Importantly, the occurrence of a large number of chiggers in residential sites warrants caution against scrub typhus infection.

As previously reported (21–23), we also observed a positive association between body size and ectoparasite loads, with larger rodents generally carrying more chiggers (Figure 4B). However, it should be stressed that this study only included island and not mainland populations. Whether chigger load will increase with subsequent gigantism when rodents colonize islands can thus not be validated here. It has been corroborated that the skull sizes of *R. tanezumi* on Orchid Island are significantly larger than those of populations in mainland Taiwan (61). The next step is to verify whether the increased body size is accompanied by a higher chigger load following colonization.

Likewise, we found more chiggers in sites with more rodents (Figure 4A). Again, this does not necessarily mean that such a positive association will hold when the rodent population increases from the mainland to the island. To our knowledge, no estimation of population density or trapping success of *R. tanezumi* in mainland Taiwan has ever been reported. Any valid comparison would further be confounded by the type of traps used, as evidenced by the detection of a much smaller rodent population when using Sherman instead of mesh traps in this study. Nevertheless, the reality that chigger abundance can increase with rodent abundance indicates that a comparison of rodent abundance and chigger abundance between mainland and island populations is a promising and worthwhile effort.

It has been observed that exotic animals are less frequently parasitized and also parasitized with fewer species in their introduced vs. native ranges (62). A few mechanisms have been proposed to explain this phenomenon. Since the distribution of parasites among hosts is highly aggregated, with most individuals being lightly parasitized (the so-called 20/80 rule) (63), most native parasites will be left behind in introduced animals. For native parasites with a complex life cycle, introduced regions might lack the hosts necessary to complete their life cycle; alternatively, the parasites of introduced regions might be host specific or have not yet evolved to utilize newly introduced hosts (62). However, the *R. tanezumi* trapped in this study were all infested with chiggers (prevalence = 100%); some individuals were even parasitized with nearly 2,000 chiggers. Since the host is only required for a single life stage (larva) for trombiculid mites and the *L. deliense* chigger can utilize a very wide range of host species (64), introduced hosts can be infested with the same amount of chiggers as their native counterparts, particularly if rodents were introduced from habitats unfavorable for chiggers (e.g., paved areas on the Taiwanese mainland) to their favorable habitats (e.g., forests on Orchid Island). Unfortunately, chigger infestation on *R. tanezumi* in mainland Taiwan has never been reported, especially in areas surrounding seaports. A study of ectoparasite infestation on *R. norvegicus* (also a commensal rodent) in Kaohsiung seaport in southwestern Taiwan reported no chiggers, which suggests that few (if any) chiggers are present in the seaport area (65). The enemy release hypothesis posits that the success of invasive species can be partially attributed to them being less parasitized in invaded sites compared with their original ranges (62, 66, 67). Nevertheless, this may not hold when the parasite has a simple life cycle, is a host generalist, and is transferred from unsuitable to suitable habitats.

It is unknown whether chiggers on Orchid Island were introduced or native to Orchid Island. Rodents are the primary hosts of chiggers (41–43), including *L. deliense* (64). If chiggers are introduced, whether accompanied by *R. tanezumi* or other exotic host species, their abundance will increase following the population growth and habitat expansion of *R. tanezumi*. On the other hand, chiggers might be native and have already occurred in several habitats (i.e., forests) of Orchid Island before the introduction of *R. tanezumi*, with other small mammal species, birds, or reptiles as the hosts. As stated above, *R. tanezumi* is more likely to thrive particularly in forests than the other extant rodent species, and rodents are a more suitable host than birds and reptiles. Therefore, the number of chiggers in forests might not greatly increase until after the introduction of *R. tanezumi* although it cannot be excluded that some extinct rodent species might be abundant and have already sustained a higher number of chiggers in forests of Orchid Island in the past.

Likewise, the origin of *R. tanezumi* on Orchid Island has not been fully resolved. The island is inhabited mainly by aboriginals of Polynesian origin known as the Tao tribe (also called Yami). The Tao are suggested to be the descendants of indigenous residents from Itbayat Island in the Philippines, 145 km south of Orchid Island (Figure 2) because both speak the same language (68) although recent molecular data have shown that few genetic exchanges have occurred between the two tribes (69). Therefore, although Orchid Island is closer to Taiwan, *R. tanezumi* might have instead been introduced from Itbayat Island. Notably, this species also occurs in the Batanes Islands, which include Itbayat Island (70). However, genetic data have shown that the *R. tanezumi* population on Orchid Island is more closely related to those of mainland Taiwan than to the Philippine populations despite the Orchid Island population not recently descending from mainland Taiwan (61). Therefore, current data still support closer origin from mainland Taiwan although the time of colonization is unknown.

Our study stresses the importance of further investigations on vector-borne and zoonotic diseases on islands. For example, some common commensal species (e.g., *R. tanezumi* and *Rattus rattus*) can not only adapt to diverse environments (71–73) but also host a variety of zoonotic diseases (8, 71). They are also regular invaders on islands (4). Accordingly, the disease risks posed by their introduction to island inhabitants should be carefully evaluated.

## Data Availability Statement

The raw data supporting the conclusions of this article will be made available by the authors, without undue reservation.

## Ethics Statement

The animal study was reviewed and approved by National Taiwan Normal University Animal Care and Use Advisory Committee (permit number NTNU-106025; NTNU-106048).

## Author Contributions

C-CK and D-LW conceived and designed the study and analyzed the data and wrote the first draft. D-LW and J-KW implemented the field work. D-LW identified the chigger species. H-CS and H-JT performed the molecular analyses. D-LW, H-CS, J-KW, H-JT, and C-CK revised the manuscript. C-CK supervised and acquired funding for the study. All authors read and approved the final manuscript.

## Funding

This study was financially supported by Taiwan Ministry of Science and Technology (MOST 104-2314-B-003-002-MY3; MOST 105-2621-M-003-003) awarded to C-CK. This article was subsidized by the National Taiwan Normal University (NTNU), Taiwan, ROC. The funders had no role in the study design, data collection and analysis, decision to publish, or preparation of the manuscript.

## Conflict of Interest

The authors declare that the research was conducted in the absence of any commercial or financial relationships that could be construed as a potential conflict of interest.

## Publisher's Note

All claims expressed in this article are solely those of the authors and do not necessarily represent those of their affiliated organizations, or those of the publisher, the editors and the reviewers. Any product that may be evaluated in this article, or claim that may be made by its manufacturer, is not guaranteed or endorsed by the publisher.
