# Supplementary figures and images for: Commensal Rodent Habitat Expansion Enhances Arthropod Disease Vectors on a Tropical Volcanic Island
Source: Front Vet Sci. 2021 Oct 8;8:736216. doi: 10.3389/fvets.2021.736216 (PMC8531417; doi:10.3389/fvets.2021.736216)

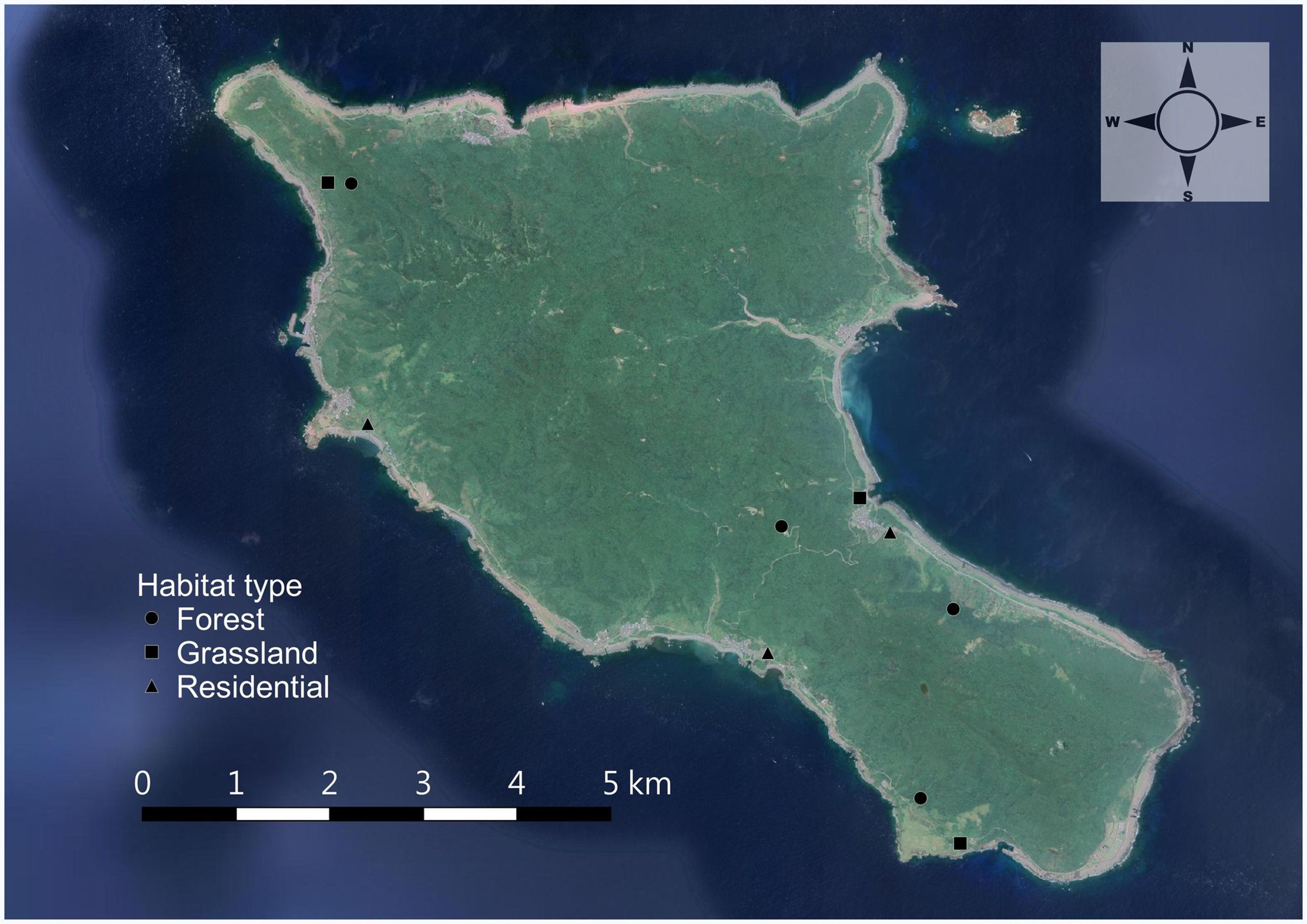

Supplement: Supplementary file 1 [file Image_1.png]
